# Application of a Coaxial-Like Sensor for Impedance Spectroscopy Measurements of Selected Low-Conductivity Liquids

**DOI:** 10.3390/s131013301

**Published:** 2013-09-30

**Authors:** Agnieszka Szypłowska, Anna Nakonieczna, Andrzej Wilczek, Bartosz Paszkowski, Grzegorz Solecki, Wojciech Skierucha

**Affiliations:** 1 The Bohdan Dobrzański Institute of Agrophysics of the Polish Academy of Sciences, ul. Doświadczalna 4, 20-290 Lublin, Poland; E-Mails: a.nakonieczna@ipan.lublin.pl (A.N.); a.wilczek@ipan.lublin.pl (A.W.); b.paszkowski@ipan.lublin.pl (B.P.); g.solecki@ipan.lublin.pl (G.S.); w.skierucha@ipan.lublin.pl (W.S.); 2 Institute of Physics, Maria Curie-Skłodowska University, Pl. M. Curie-Skłodowskiej 1, 20-031 Lublin, Poland

**Keywords:** trisodium citrate, aqueous solutions of salts, food additives, constant phase element

## Abstract

The paper presents a coaxial-like sensor operating in the 20 Hz–2 MHz frequency range used to determine the electrical properties of selected liquids of low electrical conductivity. Examined materials included low-concentrated aqueous solutions of potassium chloride, sodium chloride and trisodium citrate, which are common food additives. Impedance spectra of the measurement cell filled with particular liquids were obtained and analyzed using the electrical equivalent circuit approach. The values of physical quantities and parameters describing the equivalent circuit components, including a constant phase element, were calculated for each sample. The applied sensor was also calibrated for electrical conductivity measurements up to 8 mS/m. The constant phase element parameters differed among the studied solutions and concentrations. This may provide a basis for a detection method of small amounts of compounds, such as food additives in low-concentrated aqueous solutions. To demonstrate the potential of the presented method, samples of purchased mineral water and a flavored drink containing various additives were tested.

## 1. Introduction

Electrical measurements of the physicochemical properties of low-conductivity liquid materials are of great interest from the practical point of view. The potential areas of their application are wide and diversified, including agriculture, food industry, medicine, pharmaceutics and cosmetology. In all these fields, low concentrations of solutes are very often essential for material characteristics and play a vital role in determining whether a particular product meets the required standards. Electrical measurements can also be useful for routine laboratory analyses, which require the preservation of a sample, because they are usually fast and non-destructive. For the above reasons, they are being investigated both experimentally and theoretically [1–4].

One of the possible methods for analyzing the electrical properties of materials is impedance spectroscopy (IS), which measures the frequency spectrum of the impedance of the sample [5]. It is commonly used in the fields of electrochemistry and materials science. IS measurements provide a tool for the characterization of the structure of electrode surfaces [6], investigation of electrochemical reaction mechanisms [7], as well as various processes, like adsorption/desorption [8] or diffusion [9], taking place in the system. They are also employed in controlling the quality of metal surfaces, batteries and fuel cells [10,11]. Due to the variety and amount of information that IS may potentially provide, it is also used in biophysical [12], pharmacological [13,14] and geophysical [15] analyses.

The outcome of an IS measurement contains complex information about the electrical properties of the examined system, as well as the coupled physical, chemical and biological processes that may occur within it. For this reason, its analysis is complicated. On the other hand, an important aspect of an IS result is that it can often be related to an idealized electrical circuit composed of a set of discrete elements. Such a straightforward and widely applied manner of interpreting IS results is termed the equivalent electrical circuit (EEC) approach. An impedance spectrum of a constructed EEC reproduces the one obtained during the experiment; hence, the specific EEC may be thought of as a model of the sample being investigated [5,16]. Although fitting an appropriate EEC and providing the physicochemical interpretation of its constituents is usually ambiguous, in some cases, the types of EEC elements and the types of connections among them may suggest what kinds of phenomena or processes occur in the corresponding electrochemical system [17]. Moreover, the EEC approach provides a possibility of separating various coexisting factors and studying specific phenomena independently of others, thus facilitating the analysis and interpretation of the IS results.

A key aspect in conducting effective IS analyses of low-concentrated liquid samples is the use of an appropriately designed sensor. It should allow automated analyses to be performed in an efficient way and have an ergonomic construction. The objective of this paper is to examine the electrical properties of selected low-concentrated aqueous solutions by the IS technique using a novel coaxial-like sensor with acid-resistant steel rods. The appropriate frequency range for examining such liquids was chosen to be 20 Hz–2 MHz, as this allows the study of phenomena occurring at the surfaces of the electrodes, as well as those connected with the dielectric properties and electrical conductivity of the bulk electrolyte. This frequency range bridges the gap between the typical IS approach, which operates at as low a frequency as a fraction of a Hz, and dielectric spectroscopy techniques, which operate at frequencies from the order of MHz up to several GHz and more [18–25]. The applied frequency range is also of interest for research concerning the quality determination of foods, e.g., honey [26] and oatmeal [27], as well as in agriculture, e.g., in [28]. In the present study, the tested materials include deionized water and aqueous solutions of three compounds (trisodium citrate, sodium and potassium chlorides) ubiquitously present in food products and living organisms.

The analysis of the results obtained in the considered range of frequencies should incorporate interfacial effects. In the case of conductive liquid materials, various phenomena and processes connected with the formation and existence of an electrical double layer near the electrode surface may significantly influence the results and, thus, cannot be ignored [1,29]. The interpretation of the data was based on the EEC, which met the above requirement and was proven appropriate for the tested materials. Apart from the description of the design of the measuring sensor, the paper presents a comparison of EEC parameters obtained for different compounds and their concentrations. A calibration method for the measurement of electrical conductivity of the examined solutions with the use of the sensor is also included. The paper concludes with a demonstration of a possible application of the sensor for testing real-world mineral water and flavored drink samples and a discussion on further research opportunities. The coaxial-like geometry of the presented sensor, described in detail in the next section of the paper, was designed with a wide range of applications in mind, including measuring various inhomogeneous materials in a broad frequency range.

## 2. Materials and Methods

The tested materials consisted of deionized water with an average electrical conductivity of 0.119 mS/m and aqueous solutions of sodium chloride (NaCl), potassium chloride (KCl) and trisodium citrate. Sodium chloride is common salt, while potassium chloride (designated E508) is used for food preservation or as a sodium-free substitute for salt. Trisodium citrate is a common food additive (designated E331) used as a preservative, acidity regulator and a flavoring agent, especially in soft drinks. This compound is also used in medicine [30]. For each compound, six solutions of concentrations from 0.01 mM to 0.2 mM were measured. Additionally, nine aqueous KCl solutions of concentrations ranging from 0.01 mM to 0.5 mM were tested to provide the sensor calibration for electrical conductivity determination.

### 2.1. Experimental Setup

**Figure 1.**
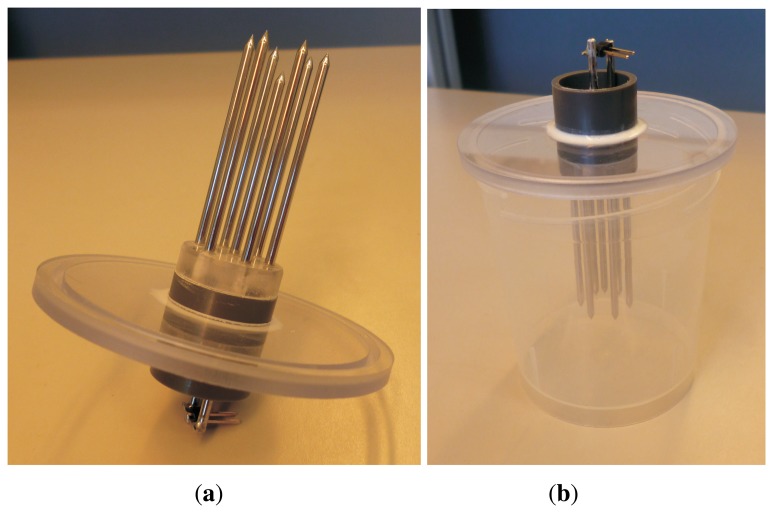
Elements of the experimental setup for impedance spectroscopy (IS) measurements. (**a**) The sensor; (**b**) a measuring cell with the sensor.

Elements of the experimental setup are presented in Figure 1. The experimental unit consisted of a sensor placed in a container with a volume of approximately 120 mL, into which the tested solutions were poured. The sensor was connected to the Agilent E4980A LCR meter (Santa Clara, CA, USA) which collected impedance readings. The LCR meter was controlled by a custom written PC application with a data logger function. The voltage signal, applied between the central rod and the outer rods of the sensor, had an amplitude of 100 mV over the frequency range from 20 Hz to 2 MHz.

The prepared solutions were placed in a climatic chamber at a temperature of 25.0 ± 0.1 °C overnight to ensure that they reached the desired temperature. Next, a given solution was poured into the measuring cell, which was placed inside the climatic chamber for at least three hours before the actual impedance measurement in order to accommodate for any possible cooling of the solution during the pouring process. To ensure the temperature stability during the measurement, the connection between the sensor and the LCR meter enabled making the measurements without opening the climatic chamber.

#### 2.1.1. Sensor Design

Homogeneous liquids can be successfully measured in a laboratory using various kinds of probes and test fixtures adapted for various electrical conductivities and frequency ranges, including capacitor cells, waveguides, transmission lines or coaxial open-ended probes. For example, in [2], an experimental cell designed for impedance spectroscopy measurements of low-conductivity liquids in the frequency range of 5 Hz to 10 MHz was presented. However, precise measurements of inhomogeneous or highly viscous materials in a broad frequency range put some special requirements on the design of a sensor. It should be easy to clean and use. The volume of the measuring cell should be possible to modify according to the demands of a given material, and the sensitivity zone of the sensor should be large enough, which is especially important for inhomogeneous materials. Moreover, it should be possible to connect the sensor to several measurement instruments—from precise LCR meters operating in low frequencies (*i.e.*, starting from the order of Hz or even below) to vector network analyzers, which operate up to microwave frequencies. This would enable performing measurements with the same sensor and on the same sample volume in a very broad frequency range with several measurement techniques, from impedance spectroscopy in lower frequencies, which was used in this study, to the frequency domain reflectometry (FDR) or the time-domain reflectometry (TDR) dielectric spectroscopy techniques, which operate in radio and microwave frequencies.

The sensor was made of seven acid-resistant steel, 2 mm diameter rods. Six of the rods were equally spaced around the seventh, centrally located one, and connected outside the measurement cell comprising a single electrode. The distance between the central rod and each of the outer rods, as well as between any two neighboring outer rods, was 7 mm. The length of the outer rods was 40 mm, while the central rod was 5 mm shorter. This ensured that in the applied frequency range, the leakage of the electric field outside of the space encompassed by the rods was minimal. The rods were placed in a polycarbonate disc inserted into a PVC tube, which passed through a polycarbonate cover. The cover was designed to form a tight seal for the measuring cell, into which the tested samples were poured. Before the measurements were performed, the sensor rods were polished and passivated by immersion in a 10% (by weight) citric acid solution at 50 °C for 30 min [31]. This treatment was necessary to enhance corrosion resistance and ensure the repeatability of the measurement results.

In the frequency range used in this study, the sensor behaves like a capacitor and enables EEC analysis typically used in impedance spectroscopy. However, with an appropriate connector, it can also be used with a vector network analyzer to perform measurements in higher frequencies. A sensor of a similar design can be used to examine inhomogeneous materials, such as soil [32].

### 2.2. Electrical Equivalent Circuit

**Figure 2.**
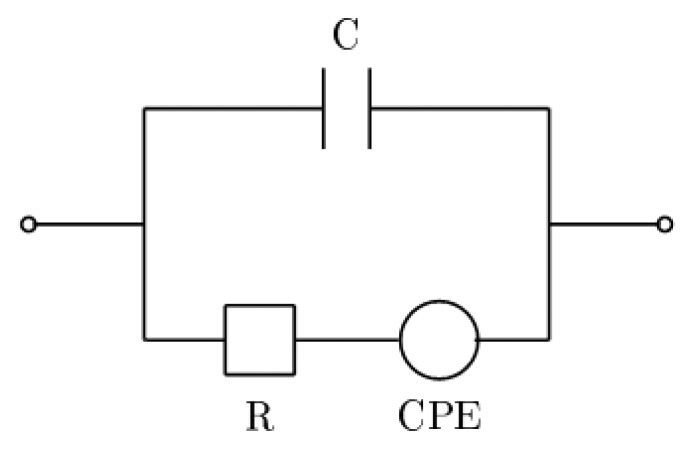
Optimal equivalent electrical circuit modeling the IS data.

The obtained results were analyzed by means of the EEC approach. The impedance spectra of various EECs were fitted to the experimental data with the use of EIS Spectrum Analyzer software [33]. It was found that in all the cases, the optimal version of an EEC was a series combination of a resistor (*R*) and a constant phase element (CPE) connected in parallel to a capacitor (*C*). The scheme is depicted in Figure 2.

The capacitor represented the total capacitance of the sensor immersed in a sample under test. The resistance, as shown in the next section, was related to the electrical conductivity of the electrolyte. The CPE described the interfacial effects on the boundary between the liquid and the electrode, such as the formation of an electrical double layer. The impedance of a CPE element depends on two parameters, namely, the dimensionless *n* and *Q* measured in units s^n^ · Ω ^−1^, according to the following relation:

(1) $$Z_{\text{CPE}}=\frac{1}{Q\;{\left(2\pi \;j\;f\right)}^{n}}$$

where $j=\sqrt{-1}$ and *f* is the frequency of the electric field. The interpretation of the CPE depends on the value of the exponent, *n*; namely, for *n* = 1, the CPE is an ideal capacitor, and for *n* = 0, it represents a resistor, while in the case of *n* = −1, the CPE becomes an inductor. If *n* is close to one, the CPE represents a lossy capacitor.

In addition to the impedance measurements, electrical conductivity of each of the tested solutions was measured independently by a Radiometer Analytical CDM210 conductivity meter (Villeurbanne Cedex, France) for reference purposes and calibration of the designed sensor for conductivity determinations.

## 3. Results and Discussion

**Figure 3.**
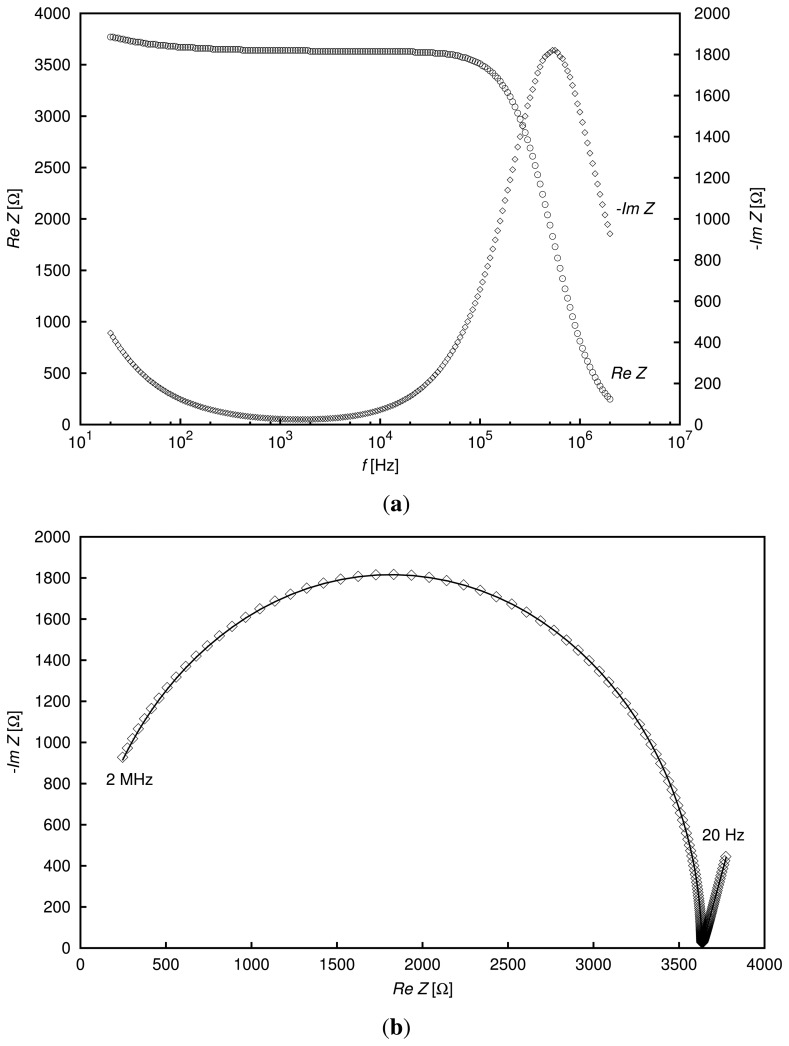
Frequency spectrum of the complex impedance of the sensor inserted into the 0.15 mM KCl solution; (**a**) real and imaginary parts of impedance as functions of frequency; (**b**) Nyquist diagram. Points represent the measured experimental spectrum; the continuous line depicts the impedance spectrum calculated from the determined equivalent electrical circuit (EEC) parameters.

The impedance spectrum obtained from a measurement of the 0.15 mM potassium chloride solution is shown in Figure 3. On the top plot of the figure, the real and imaginary parts of the impedance as measured by the LCR meter are shown as functions of frequency. One can compare this spectrum to the results reported in [2], where the KCl aqueous solution of a concentration of 0.6 mM was examined in the frequency range 5 Hz–10 MHz. As can be seen, even in a narrower frequency range, which was applied in the present study, all relevant features of the spectrum are evident, including the impact of the electrode-electrolyte interface impedance modeled in this work by the constant phase element. This effect, visible in the low-frequency part of the spectrum as an increase in the real part of the impedance, *Z*, is even more conspicuous for higher concentrations of the solutions. The relaxation frequency also increases with the increase in concentration. On the bottom plot of Figure 3, there is the Nyquist diagram of the same measured impedance spectrum in the complex impedance plane, as well as the impedance obtained from fitting the EEC spectrum to the experimental data. As may be inferred from the diagram, the chosen EEC was appropriate for modeling the electrical properties of the examined sensor-liquid system for the considered frequency range. The straight line fragment on the Nyquist diagram at low frequencies depended on the CPE parameters.

**Figure 4.**
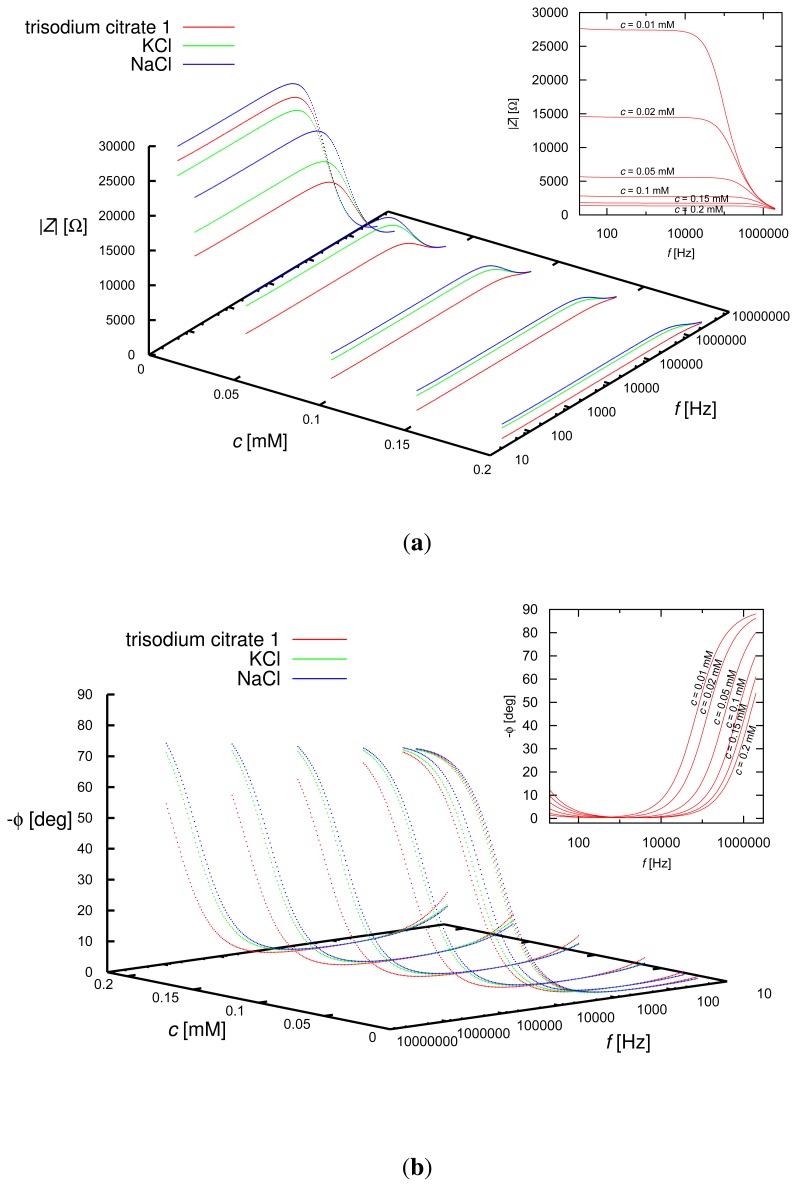
(**a**) Magnitude and (**b**) phase of the complex impedance, *Z*, of the sensor inserted into KCl, NaCl and trisodium citrate (series 1) solutions of various concentrations, *c*, as functions of frequency, *f*. The plots in the right upper corners depict the dependence of the magnitude and phase of the impedance on the concentration of the trisodium citrate solutions.

The frequency dependence of the magnitude and phase of the impedance, *Z*, of the sensor-liquid system for the KCl, NaCl and trisodium citrate solutions of various concentrations are shown in Figure 4. The dynamic range of the presented system is determined by the capabilities of the LCR meter, as well as the total capacitance of the measuring cell, which, in turn, depends on the geometry of the sensor. As can be inferred from the plots, both the magnitude and phase of the impedance depended on the concentration of the solutions, as well as on the type of the solute. The differences among the tested solutions were evident also for the electrical equivalent circuit parameters described in the next section.

### 3.1. EEC Parameters

**Figure 5.**
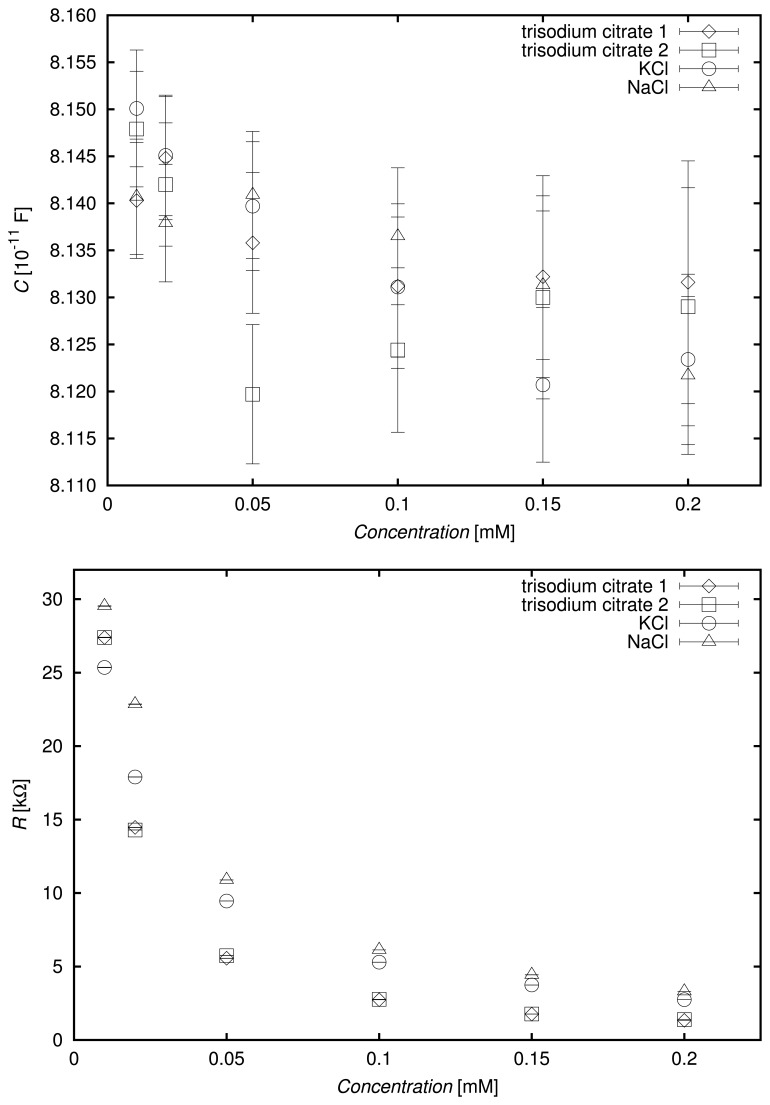
Determined values of the capacitance (*C*) and resistance (*R*) of the EEC elements for the examined solutions at different concentrations.

**Figure 6.**
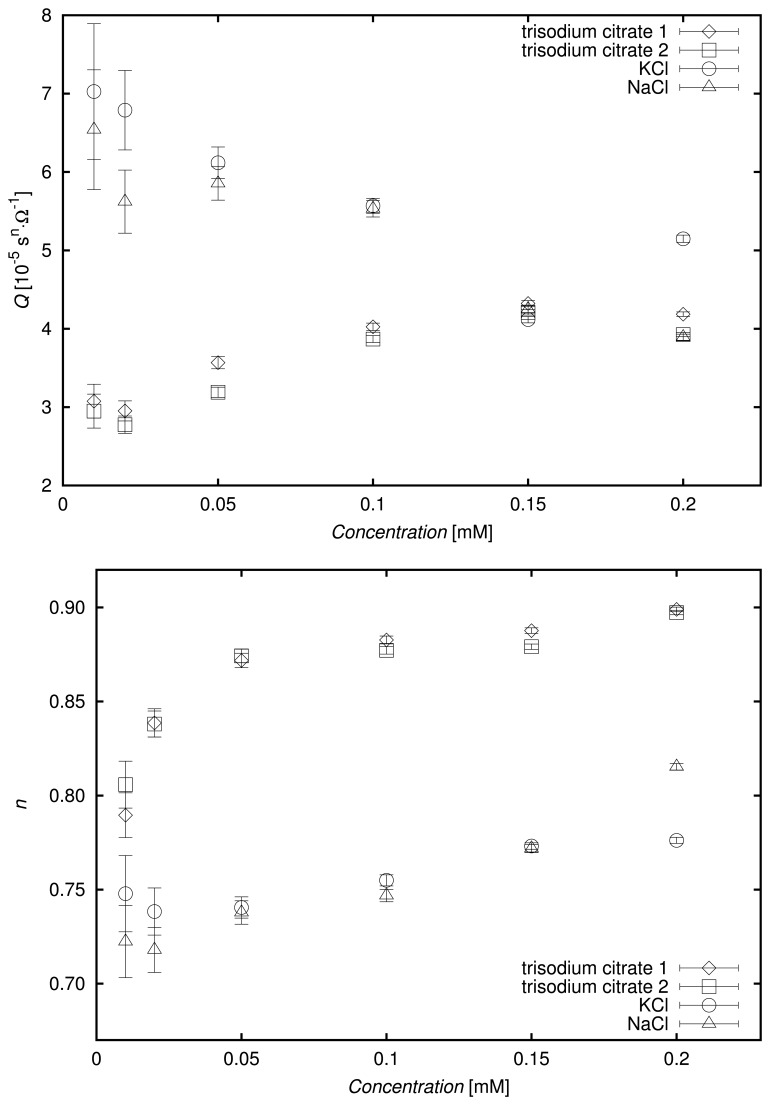
Determined values of the constant phase element (CPE) parameters for the tested solutions at different concentrations.

Figures 5 and 6 present the obtained values of parameters *C*, *R*, *Q* and *n* of the fitted EEC for the tested aqueous solutions of potassium and sodium chlorides and trisodium citrate of concentrations: 0.01, 0.02, 0.05, 0.1, 0.15 and 0.2 mM. Each of the trisodium citrate solutions was tested twice, exhibiting a very good repeatability in the results. The error bars in the figures represent standard errors of the determined EEC parameter values calculated using the EIS Spectrum Analyzer software [33].

The capacitance values did not vary much from the capacitance obtained for deionized water (not shown in the graphs), due to the low concentrations of the solutions. In general, it may be stated that the calculated capacitance tended to decrease as the concentration increased. This might be related to the effect of the decreasing dielectric permittivity of the medium, due to increasing formation of ionic hydration shells. However, the standard error of determination of the parameter, *C*, of the EEC increased for highly concentrated solutions characterized by high electrical conductivity.

The resistance of the resistor in the EEC decreased as the concentration of solution increased for all tested compounds, as expected. Moreover, the resistance values for a particular concentration were in increasing order according to the following compound sequence: trisodium citrate–potassium chloride–sodium chloride. This relation was arguable at low concentrations, due to significant errors connected with the calculated values.

The parameters, *Q* and *n*, used to characterize the constant phase element should be interpreted simultaneously, because the unit of *Q* depends on the value of *n*. However, due to the low variability of *n* for all concentrations of a particular compound, some general conclusions may be drawn. The values of *Q* increased with increases in concentration for trisodium citrate. Conversely, they decreased when the amount of solute increased in the aqueous solutions of potassium and sodium chlorides. Due to the relatively high standard error of determination for *Q* at low concentrations, these statements should be tested using larger numbers of measurement repetitions and appropriate statistical methods, which is the goal of a separate study.

The value of *n* increased when the concentration increased for all the investigated chemical compounds. Very good agreement was observed for two independent series of trisodium citrate solution measurements, and the values of *n* were similar for all concentrations of the aqueous solutions of potassium and sodium chlorides. With the exception of the lowest concentrations, for which large standard errors were obtained, the values of *n* for trisodium citrate were significantly different from those of the chlorides.

The above results may indicate that the values of the parameters characterizing the constant phase element are strongly dependent on the types of ions present in the solution, which may be particularly related to the masses and spatial structures of the ions. A detailed analysis of the constant phase element for aqueous solutions of a wide range of food additives, including preservatives and flavoring agents, will be a subject of a separate study.

### 3.2. Electrical Conductivity Measurement

**Figure 7.**
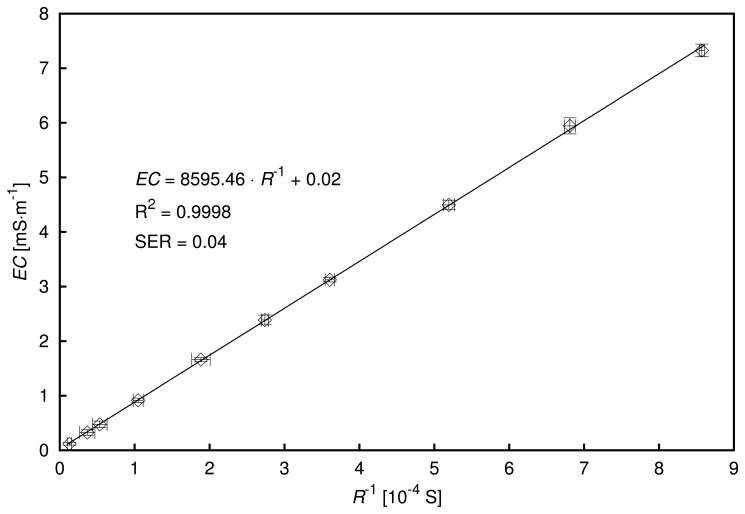
Electrical conductivity (*EC*) of deionized water and KCl solutions measured using a conductometer *versus* the reciprocal of the resistance, *R*^−1^, of the EEC element.

Because the constant phase element parameters depend on the concentration of the solution, a precise determination of the electrical conductivity by the presented sensor is very important in the context of its possible application for detecting small amounts of chemical compounds and differentiating among them. The relation between electrical conductivities of deionized water and potassium chloride aqueous solutions of concentrations of 0.01, 0.02, 0.05, 0.1, 0.15, 0.2, 0.3, 0.4 and 0.5 mM, as measured with a conductometer, and the reciprocals of their resistances, *R*^−1^, calculated during the EEC fitting. is shown in Figure 7.

The conductivity readings and impedance spectra collections were performed three times for each tested solution, and the average conductivity was calculated, as well as the average reciprocal of resistance. The error bars refer to expanded uncertainties, which are combined measurement uncertainties multiplied by a coverage factor, which was chosen for the Student's *t*-distribution with a confidence level of 95%. The combined conductivity measurement uncertainty was calculated as a square root of the sum of uncertainties of type A and B squared. The uncertainty corresponding to random errors was estimated on the basis of the standard deviation of the average value of conductivity. On the other hand, the uncertainty connected with systematic errors was equal to the limiting error, Δ*C_syst_*, divided by $\sqrt{3}$ based on the assumption that the distribution of systematic errors was uniform. The limiting error for a particular conductivity reading, *EC*_0_, was (0.2% · *EC*_0_ + 0.003) S·m^−1^, according to the specification of the conductometer. The combined measurement uncertainty of the reciprocal of *R* was the standard deviation of its average. As can be seen in Figure 7, the errors for electrical conductivity and *R*^−1^ were very low.

The obtained dependence of the measured electrical conductivity on *R*^−1^ was linear with a very high value of the coefficient of determination, R^2^, of 0:9998 and a standard error of regression, SER, of 0:04. This outcome proved that the designed sensor can be also used for accurate conductivity measurements and that the EEC was selected appropriately for the tested solutions. The calibration function for determining the electrical conductivity of low-concentrated solutions of interest is given in Figure 7.

### 3.3. Detection of Additives in Mineral Water—A Possible Application

To test the presented sensor and the measurement method for real-world products analysis, samples of a still mineral water of a popular brand and a strawberry flavored drink based on the same mineral water purchased in a local supermarket were examined. The total amount of mineral salts present in the water was equal to 650 mg/L, as specified by the producer. The amounts of the dominant ions in the water provided on the bottle label are presented in Table 1.

The examined flavored drink consisted of 96% of the same mineral water, according to the producer specification, and contained a number of various food additives, including glucose-fructose syrup, sugar, citric acid, natural flavors and artificial sweeteners—acesulfame K and sucralose. To lower the electrical conductivity of the examined products to the values measurable by the presented sensor, the samples under test were diluted 1:20 in distilled water. These samples were then tested in three repetitions each, and the parameters of the EEC presented in Figure 2 were determined. In order to determine whether the observed differences in EEC parameters between the mineral water and the flavored drink samples could be explained just by the difference in their electrical conductivities, a third set of samples was prepared by diluting the mineral water 1:10 in distilled water. The values of the electrical conductivity of these samples approximated that of the flavored drink diluted 1:20.

**Figure 8.**
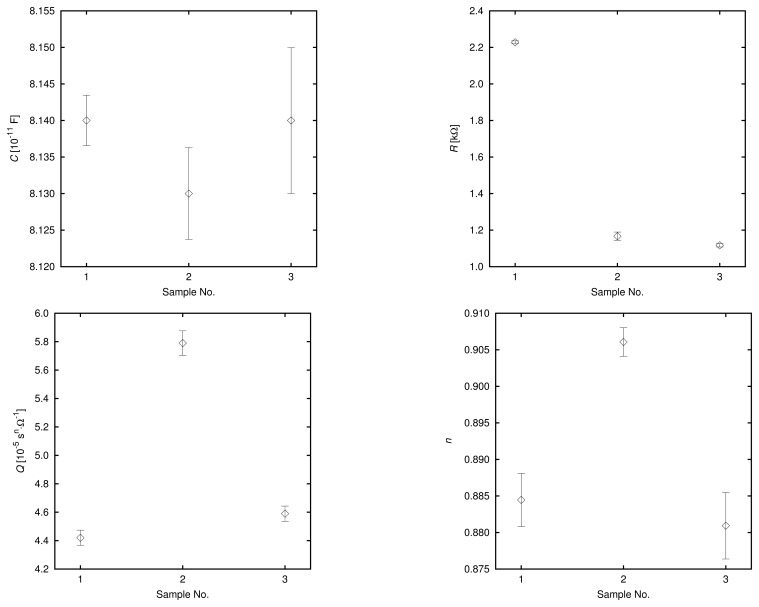
EEC parameters of mineral water diluted 1:20 (sample no. 1), flavored mineral water diluted 1:20 (sample no. 2) and mineral water diluted 1:10 (sample no. 3).

The obtained EEC parameters, *C*, *R*, *Q* and *n*, for the tested samples are presented in Figure 8. Each point represents an arithmetic mean over three repetitions, and the error bars correspond to the 95% confidence intervals. As expected, the capacitance did not vary significantly among the tested cases. The value of the resistance, *R*, is significantly higher for the mineral water diluted 1:20 (sample no. 1 on the graphs) than for the flavored drink diluted 1:20 (sample no. 2) and mineral water diluted 1:10 (sample no. 3). This was due to the different electrical conductivities of the samples. Both of the CPE parameters for the flavored drink were significantly different than for mineral water samples. Since the values of these parameters for mineral water diluted 1:10 were very close to those obtained for mineral water diluted 1:20, it can be stated that the observed differences were caused by the presence of the additives in the flavored drink and cannot be explained only by the difference in the concentration of the basic ions.

The obtained EEC parameters were very stable over the measured samples. The relative standard deviations were lower than 0.05%, 0.6%, 0.5% and 0.2% for capacitance, *C*, resistance, *R*, parameter *Q* and parameter n, respectively.

## 4. Conclusions

The paper presented a coaxial-like sensor with acid-resistant steel rods used for the examination of low-conductivity liquids. The electrical properties of chosen low-concentrated aqueous salt solutions were examined using impedance spectroscopy methods within the frequency range from 20 Hz to 2 MHz, which was appropriate for the investigated samples.

The selected electrical equivalent circuit modeling the impedance of the sensor-electrolyte system was appropriate for the measured cases. The values of the resistance, *R*, depended strongly on the electrical conductivity of the bulk electrolyte—the reciprocal of *R* can be used to calculate the electrical conductivity of a tested liquid with the obtained calibration function. The presented sensor can be used to measure the conductivities of liquid materials up to 8 mS/m, which is sufficient for the solutions of interest. This value is limited by the frequency range—the fragment of a semicircle on the Nyquist plot for high-concentrated solutions is too small for an accurate fit of the EEC parameters. Therefore, to measure electrolytes of higher conductivities, frequencies of the electric field of the order of MHz and above should be employed. The lowest and highest measurable concentrations were determined by the accuracy of the EEC fitting, which depended on the electrical conductivity of the tested liquid. The highest accuracy in the determination of the EEC parameters was observed for solutions of a concentration not lower than 0.05 mM in the case of the KCl and NaCl and not lower than 0.02 mM for the trisodium citrate, with the upper concentration limit equal to 0.5 and 0.2 mM for KCl and trisodium citrate, respectively. The coaxial-like geometry of the sensor enables, after necessary connector modification, one to perform measurements with the use of a vector network analyzer in a frequency range high enough to examine even highly conductive food products, such as fruit juices, which is the subject of a separate study currently in progress [34].

The constant phase element of the EEC depended on the type of the solute. Therefore, during further research, it is worth investigating whether it is possible to develop a ‘fingerprint’ library of the electrical properties of various chemical compounds, which may become the basis of a quick and non-destructive method of detecting which chemical compound is present in a given solution. The tested mineral water contained a composition of a multitude of ions, and the flavored drink contained also various additives. The obtained significant differences among the EEC parameters (especially the CPE parameters, *Q* and *n*), which are not explainable solely by the differences in conductivities, provide motivation for the analyses of mixtures of various food additives and other compounds present in food. For this reason, the obtained results are promising in the context of future applications for quality determination and control of liquid materials of agricultural and biological origin, including detection of preservatives and other additives in various types of foods, such as drinks and dairy products.

## Figures and Tables

**Table 1.** The concentration of the dominant ions present in the tested mineral water, as specified on the bottle label.

**Ion**	${\text{HCO}}_{3}^{-}$	**Cl**^−^	**F**^−^	**Ca**^2+^	**Mg**^2+^	**Na**^+^	**K**^+^
**Concentration (mg/L)**	448.1	12.6	0.3	114.2	20.0	10.0	2.5
